# Efficiency of the traditional practice of traps to stimulate black truffle production, and its ecological mechanisms

**DOI:** 10.1038/s41598-022-19962-3

**Published:** 2022-09-28

**Authors:** E. Taschen, G. Callot, P. Savary, M. Sauve, Y. Penuelas-samaniego, F. Rousset, X. Parlade, M.-A. Selosse, F. Richard

**Affiliations:** 1 Eco & Sols, Univ Montpellier, CIRAD, INRAE, Institut Agro, IRD, Montpellier, France; 2 26 chemin des olivettes, 34980 Montferrier sur Lez, France; 3 Rue des Champs, La Remisière, 17480 Le Château d’Oléron, France; 4 CEFE UMR 5175, CNRS - Université de Montpellier - Université Paul-Valéry Montpellier – EPHE, 1919 Route de Mende, 34293 Montpellier, France; 5 ISEM CNRS UMR 5554, Université de Montpellier, CNRS, IRD, EPHE, CC 065, Place Eugène Bataillon, 34095 Montpellier, France; 6 Mycorrhizas-Sustainable Plant Protection, IRTA, Ctra. de Cabrils, 08348 Cabrils (Barcelona), Spain; 7 UMR 7205 ISYEB, Institut Systématique Evolution Biodiversité, Muséum National d’Histoire Naturelle, CNRS, Sorbonne Université, CP 50, 45 rue Buffon, 75005 Paris, France; 8 Department of Plant Taxonomy and Nature Conservation, University of Gdansk, Wita Stwosza 59, 80-308 Gdansk, Poland

**Keywords:** Ecosystem ecology, Molecular ecology, Population dynamics, Plant ecology

## Abstract

The black truffle *Tuber melanosporum* was disseminated all over the world, propelled by the development of a wide variety of empirical practices. A widespread practice, called ‘truffle trap’, consists of placing pieces of truffles into excavations dug under host trees, and of collecting truffle in these traps in the next years. This research aims at (1) evaluating the effect of this practice on fruitbody production based on the analysis of 9924 truffle traps installed in 11 orchards across *T. melanosporum* native area in France and (2) exploring the mechanisms involved in fruitbody emergence using traps where the genotypes of introduced truffles were compared with those of fruitbodies collected in the same traps. We confirmed that truffle traps provide a major and highly variable part of truffle ground production, representing up to 89% of the collected fruitbodies. We evidenced a genetic link between introduced spores and collected fruitbodies, and then demonstrated that truffle growers provide paternal partners for mating with local maternal mycelia. We also highlighted that soil disturbance stimulate the vegetative development of established maternal mycelia. This research supports that a widely used traditional practice enhances fruitbody production by shaping favorable conditions and providing sexual partners required for fruiting.

## Introduction

Understanding the ecological and biological bases of the traditional agricultural practices is a major challenge shared by anthropologists, biologists and ecologists^1^, but also a source of modern innovation to develop sustainable agricultural systems^2^. Practiced in Europe, and secondarily in many parts of the world today, black truffle (*Tuber melanosporum*) cultivation is concentrated in planted orchards, where truffle production is intensified under inoculated trees^3^. However, the cultivation of truffles is still far to be fully controlled. Modern methods such as *T. melanosporum* inoculation of tree seedlings in nursery, summer irrigation, plastic mulching and mechanical tillage coexist with empirical practices often inspired from the observation of natural ecosystems^3,4^. The most recurrent of these empirical practices consists of the yearly dispersion of crushed black truffle fruitbodies on truffle grounds, at the time of plantation and later, considered as a way to imitate regular deposition of spores by animal feces. The first evidence of empirical dispersion of crushed fruitbodies dates back to 1564^5^. Nowadays, the practice evolved into a widespread usage across the natural range of *T. melanosporum*, in Spain, France and Italy, which consists of placing fungal material into small excavations dug under truffle host trees: the so-called ‘*spore traps’*, ‘*Catalan holes’*^6^, ‘*truffle nests’*^7^ or hereafter *‘truffle traps’*. These designs vary in the dimensions of excavations, their refilling substrate, and the amount of fungal material added, and in the level of preservation of the host’s root system^6,8^. Yet, all designs are based on placing pieces of truffles in soil (*e.g*.^9,10^; see^11^ for a recent review), and target intensification and acceleration of truffle production. Recently, first studies from geographically restricted area provided evidence that truffle traps may shape morphological traits of fruitbodies and drive reduced interaction with highly damaging mycophagous insects^7^, and may sustain an increased fruitbody production^6,7^. However, the mechanisms underlying the global ‘trap effect’ remain unclear.

The black truffle is a heterothallic ascomycete whose sexual life has been recently clarified^12–14^. Truffle reproduction requires a mating event between two haploid individuals leading to a transitory diploid zygote that immediately undergoes meiosis and produce numerous haploid spores enclosed in a hypogeous fleshy fruitbody, called the ascocarp (or, commercially, the truffle^13,15–18^. In the same way as other ectomycorrhizal fungi, the black truffle associates with roots of a wide range of trees^19^ from which carbohydrates are derived to support the development of soil mycelia^20^ and the edification of ascocarps^21^.

**Figure 1:**
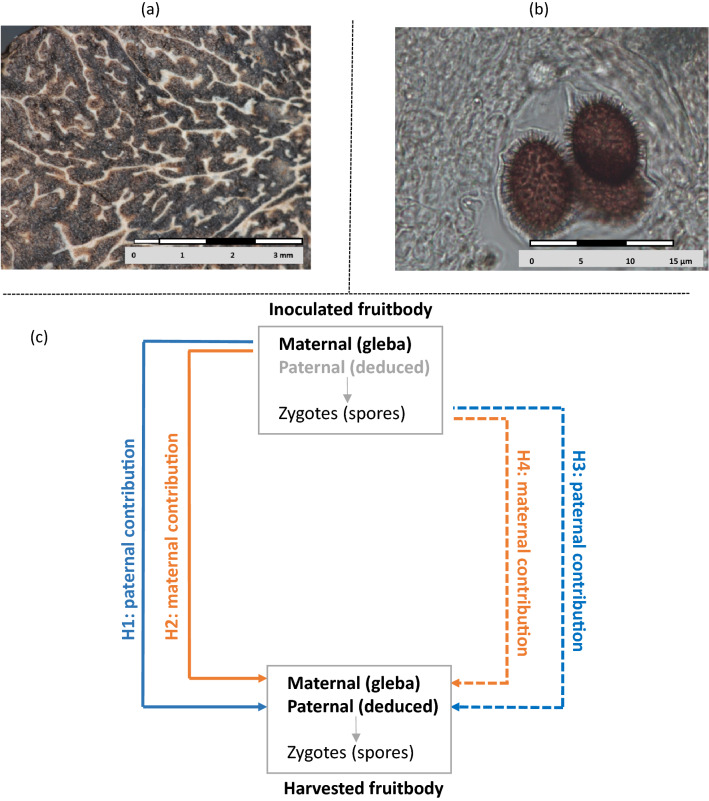
(**a**) Cut into a fresh *T. melanosporum* fruitbody showing the gleba (white flesh) which is the maternal individual, and the regions containing the meiotic spores (brownish parts). Photo credit G. Callot (**b**) Detailed view of *T. melanosporum* ascus containing spores under optical microscope. Photo credit F. Richard (**c**) Theoretical scenarii and corresponding hypotheses of the genetic contribution of *T. melanosporum* crushed fruitbody inoculum to the production of fruitbodies in truffle traps. Hypotheses H1 and H2 concern the potential contribution of the gleba from the inoculated fruitbody (continuous lines) to the harvested fruitbodies and hypotheses H3 and H4 concern the potential contribution of spores from the inoculated truffle (dotted lines). Both the gleba and the spores can have either a paternal contribution (blue) or maternal contribution (orange) to the harvested fruitbody. Hypotheses H1 and H2 were tested by direct multilocus genotype comparisons whereas hypotheses H3 and H4 were tested by relatedness estimations.

Only one parent (considered as the maternal one) is perennially established as ectomycorrhizal, and this connection allows it to invest in ascocarp development and spore protection, by forming the ascocarp flesh, the so-called gleba (Fig. 1a). The second individual (considered as paternal) is only detected by its genetic contribution to meiotic spores (Fig. 1b) encased in the produced fruitbodies^18,22^. The biology of this second partner remains unclear as it is never detected on roots of nearby plants^23,24^. Its temporal transience and the reduced area it occupies in soils suggests an existence reduced to ephemeral germlings from the soil spore bank^18,22,25^. In planted orchards and spontaneous truffle grounds, it has been observed that (1) each individual can play either a maternal or paternal role, whatever the mating type it carries (*i.e.* MAT1-1 or MAT 1-2 mating type allele^16^, the only condition being to be established as ectomycorrhizal on host roots to play a maternal role^13,18,22^, and (2) a hitherto unexplained spatial segregation of clusters of individuals of same mating types, possibly due to a cooperation between related individuals in soils^17,18,26^. The full understanding of the sexual reproduction of the black truffle is still a main lock to better adjust cultivation practices.

The success of truffle traps, a practice which developed and spread across networks of truffle growers during the last two decades, relies on expected enhanced yields. First, this practice may reduce the time to produce truffles in orchards, currently between 8 and 15 years^27^, to 5 or 6 years when truffle traps are prepared^11^. Second, truffle traps are reported to increase production of truffles two years after their setting up^6,8,11^. Indeed, more generally, the dispersion of crushed truffle as a method to produce truffles has been repeatedly reported during the last centuries by botanists^9,28^, foresters^29^, physicians^30,31^ and agronomists (*e.g.*^8^, although sometimes with skepticism^8,32^. However, the success of such inoculations has never been scientifically demonstrated, and much less explained on the basis of the involved mechanisms: do the spores contribute to fruitbody production in a male and/or a female way? The inoculation at precise location in truffle traps offers unique opportunities to track the inoculum.

Using a two-step analysis, the present study aims at (1) elucidating the biological and ecological bases of truffle traps effect on fruitbody production, and (2) evaluating the efficiency of adding crushed truffle material to assist the mating of the black truffle. First, this study reports fruitbody production recorded in eleven truffle orchards from southwestern France, inside and outside 9924 truffle traps installed from 2004 to 2012 under 1080 oaks. Using this dataset, three orchards with high within-trap production were selected for an in situ experiment. Truffle traps are a complex practice combining inoculum manipulation (addition of crushed fruitbodies) and small-scale soil disturbance (excavation and refilling). Our experimental design enabled us to evaluate the contribution, on the vegetative development of *T. melanosporum* mycelium and fruitbody production, of soil disturbance only (hereafter, *disturbance effect*) *versus* the effect of adding crushed truffle material (hereafter, *inoculum effect*). To investigate the genetic contribution of inoculants on fruitbody production, we analyzed the genetic relatedness between inoculants and truffles gathered in truffle traps over two years after their settlement. We separately genotyped the paternal and maternal genotypes of (1) truffles used as inoculum, and (2) truffles collected in truffle traps two years later to test four hypothetical contributions, *i.e.* the gleba or the spores of the inoculum each playing either a paternal and/or a maternal role in the formation of harvested truffles (H1 to H4 in Fig. 1c).

## Results

### Analysis of truffle growers’ archives (Dataset 1)

**Figure 2:**
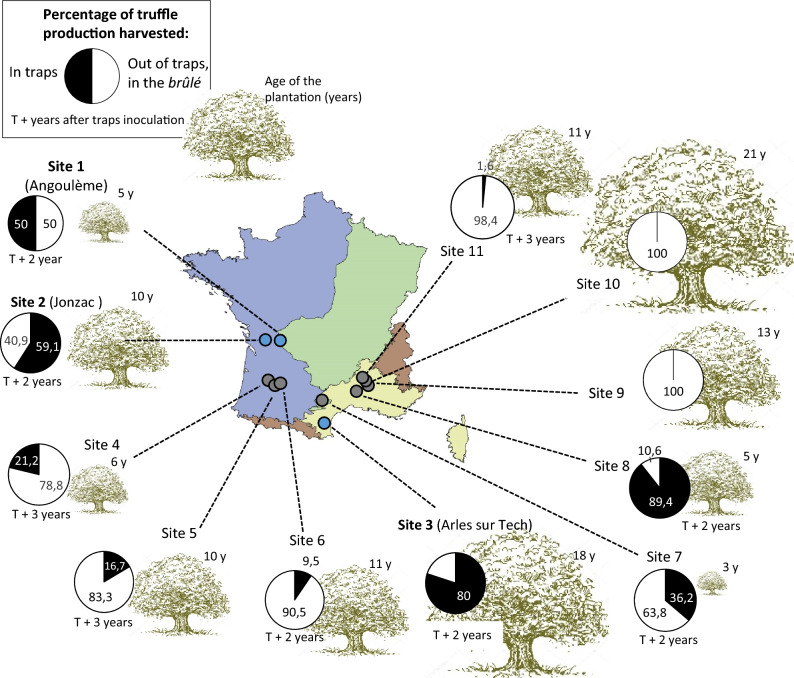
Geographical distribution of the study sites, with indication of the proportion of truffle biomass harvested inside truffle traps (in black) and in the surrounding *brûlés* (in white), expressed in percentage. Numbers in tree symbols indicate tree age (top, which is also reflected by the size of the tree) and the number of years needed to gather the first truffles in truffle traps (bottom). Colors in the map indicate French climatic domains as follows: yellow: Mediterranean, blue: Oceanic, green: continental and brown: mountain, from Noirfalise, A.. Map of the Natural Vegetation of the Member Countries of the European Community and the Council of Europe: Scale 1: 3.000. 000 (Vol. 10,970). Office for Official Publications of the European Communities (1987). Map freely accessible at https://inpn.mnhn.fr.

The analysis of fruitbody production was performed on data collected between 2004 and 2016 by 11 truffle growers: overall, they designed 9924 truffle traps under 1080 oaks (Table S1). Truffle harvesting occurred two years after inoculum in a majority of analyzed orchards (*i.e*. in 6 out of 11; see Figure S1 for examples at Site 2 and Site 8), and more rarely three years after (at three orchards; Fig. 2).

At the scale of truffle orchard, the proportion of fruitbody produced in traps ranged from 0 (Sites 9 and 10) to 89.4% (Site 8) of the whole recorded production of the site (from 4 to up to 16 years-long recording period; Fig. 2). These contributions originated *from* cumulated surfaces of truffle traps rangin*g fr*om 0.14 to 2.65% of the total productive area of the orchards (Table S1). For instance, at Site 2, the proportion of fruitbodies collected between 2010 and 2015 within 72 truffle traps installed under 36 trees, *i.e.* in 0.14% of the total area of brûlés, averaged 59.1 ± 6.6% of the total production of the orchard (Table S1). In summary, the contribution of truffle traps to the total production of the orchard was null in two of them, low (*i.e.*, less than 10%) in two others, minority (*i.e.*, between 10 and 50%) in three others, and majority in the four last ones, where more than 50% of fruitbodies were collected in approximatively 0.7% of the productive surface of the orchard (mean percentage of truffle trap surface on cumulated surface of *brûlés* per orchard; Fig. 2 and Table S1).

### Analysis of fruitbody production and mycelium concentration in experimental truffle traps (Dataset 2)

**Figure 3:**
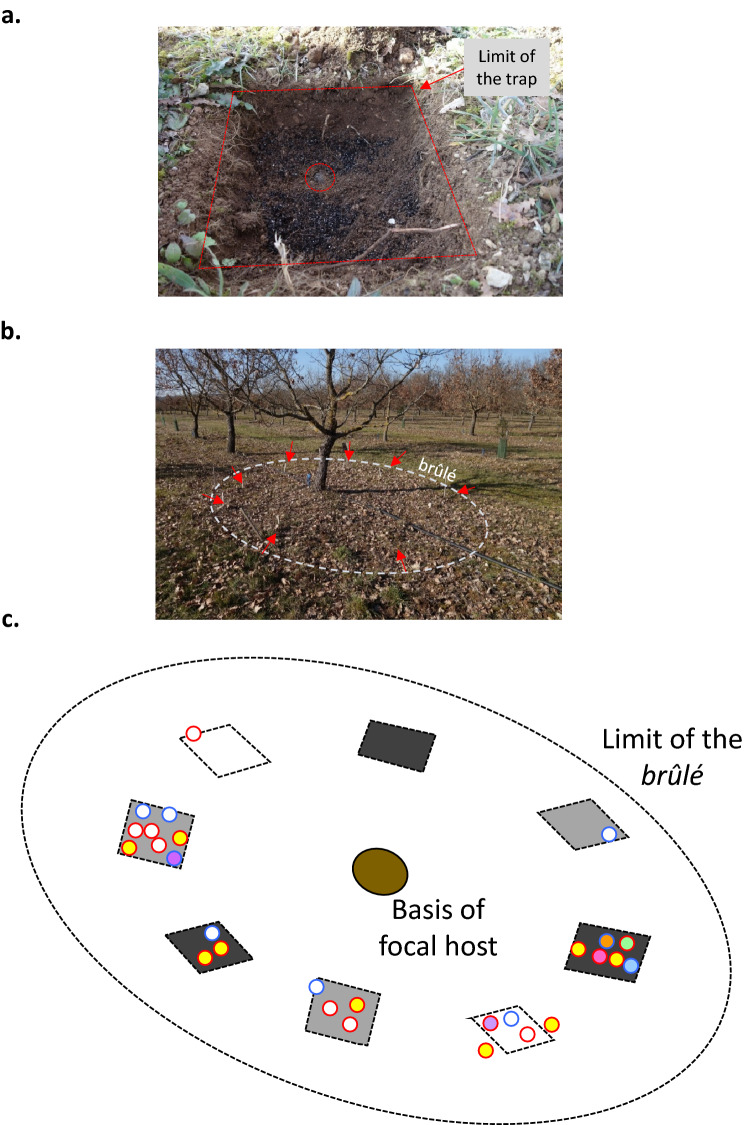
(**a**) View of a truffle trap at final collecting time, with indication of the limit of the trap (dotted white line) and the position of a fruitbody developed at its ground (red circle). Photo credit F. Richard (**b**) View of experimental device after two years and before collecting fruitbodies showing the position of experimental traps (red arrows) all around the host tree within the *brûlé*. Photo credit F. Richard (**c**) Schematic representation of a *brûlé* on site 2 (Jonzac), localizing harvested fruitbodies (circles) in- or outside of non-inoculated traps (white) versus traps inoculated with one mating type gleba (light grey) versus with two mating type glebas (dark grey), and distinguishing the multilocus maternal genotypes according to the color of circle inside -(white indicates non identified MLGs) and the mating type of the gleba according to the color of circle outline (maternal individual; Mat 1-1 circled in blue and Mat 1-2-1 in red). All identified paternal MLGs were different, and are not represented for easiness of reading.

At the three study sites, two years after implementing the experiment (including a third year on Site 1), 119 fruitbodies were collected under ten host trees, including 15 (12.8%) in *non-inoculated control traps*, 80 (67.2%) in *inoculated traps* and 24 (20.2%) in the surrounding brûlés (Table S2, and Fig. 3c for an example of production at Site 2). It is noteworthy that over the three sites, 68.4% of harvested truffles were not detected by trained dogs during the systematic survey, but only discovered when all traps were excavated at experience ending. Among the 95 fruitbodies collected in traps, 45 developed not within the introduced substrate, but at the interface with the undisturbed soils surrounding the trap (< 1 cm of the limit between disturbed and undisturbed soil).

Over the three sites, the production in *non-inoculated control traps* (25, 25 and 9.38 fruitbodies/m^2^ in average at Site 1, Site 2 and Site 3; Table S2) did not significantly differ from that in *inoculated traps* (37.5, 37.5 and 27.08 fruitbodies/m^2^ in average at Site 1, Site 2 and Site 3). The density of truffles was at best slightly higher (1.78-fold, *p*-value = 0.59 by likelihood ratio test) in the *inoculated traps* than in the *non-inoculated control traps*. No difference was apparent between traps inoculated with one *versus* two mating types (in particular, no higher density with two mating types). The fruitbody density was fourty-eight fold higher in the *non-inoculated control traps* than in surrounding brûlés (*p*-value < 1e-4 by likelihood ratio test; Table S3).

The concentration of *T. melanosporum* mycelium in soil varied significantly across sites with mycelium at Site 2 (Jonzac) being less concentrated than the two others (Table 1). There was no significant difference in mycelium concentration between control traps and inoculated traps (*p*-value = 0.08 by likelihood ratio test).

**Table 1:** Mean concentrations of *T. melanosporum* mycelium in truffle traps and in soil out of *brûlés* at the three study sites.

	*T. melanosporum* mycelium concentration (μg/g soil)			
Site	Non inoculated control traps	1-gleba mating type traps	2-gleba mating type	Outside brûlés
1 (Angoulême)	1021,9	991,9	1362,9	127,3
2 (Jonzac)	13,8	12,9	5,2 b	0,2
3 (Arles sur Tech)	1397,2	840,0	332,1	8,8

### Genetic structure of fruitbody populations in truffle traps

Overall, the MLGs analysis of 58 maternal genotypes revealed 36 different MLGs (Table 2). Three of them were represented by more than two fruitbodies, leading to a clonal diversity of 0.61. Contrastingly, the MLG analysis of 49 paternal genotypes revealed 49 distinctive MLGs (Table 2), i.e. a clonal diversity of 1.

**Table 2:** Number of *T. melanosporum* fruitbodies included for genotyping and genetic analyses testing the contribution of the inoculum to the harvested fruitbodies (see the four hypotheses in Fig. 1c).

	Site 1	Site 2	Site 3
*MLG analyses*			
Nb of analysed maternal individuals	27	16	15
Nb of MLGs	22	8	6
Nb of analysed paternal individuals	19	18	12
Nb of MLGs	19	18	12
*Contribution of the gleba*			
Paternal (H1)—comparison of MLGs	no	no	no
Maternal (H2)—comparison of MLGs	no	no	no
*Contribution of the spores*			
Paternal (H3)—Relatedness *r* (*p*-value)	0.062 (0.132, ns)	− 0.041 (0.721, ns)	0.417 (0, ***)
Maternal (H4)—Relatedness *r* (*p*-value)	0.033 (0.239, ns)	0.052 (0.195, ns)	− 0.047 (0.698, ns)

***p-values < 5e- 4 after Bonferroni correction.

On each host tree included in the experiment, multiple maternal MLGs were identified, in both non-inoculated and inoculated traps. On six of the ten trees included in the experiment, and within 11 of the 22 traps containing truffles at harvest date, maternal MLGs of the two mating types were detected (see Figure S2 for an example from Site 2). Under each of the three host trees presenting more than 10 fruitbodies (Table S2), two to 15 maternal MLGs were identified (Table S4), with up to seven different maternal MLGs in a single trap (see Figs. 3c and Figure S2 for examples).

### Genetic relatedness between collected fruitbodies and inoculum introduced in truffle traps

Overall, 104 maternal and 95 paternal MLGs were successfully included in relatedness analyses. The relatedness estimates of inoculated spores to harvested maternal genomes (corresponding to the test of hypothesis H4 in Fig. 1c) were 0.033, 0.052 and − 0.047 for Site 1, Site 2 and Site 3 respectively (with *p*-values estimates from 10,000 simulated samples of 0.239, 0.195 and 0.698 at Site 1, Site 2 and Site 3 respectively; Table 2). The relatedness estimates of inoculated spores to harvested paternal genomes (corresponding to the test of hypothesis H3 in Fig. 1c) were 0.062, − 0.041 and 0.417 for Site 1, Site 2 and Site 3 respectively (Table 2). For each population, *p*-values estimates from 10,000 simulated samples were 0.1316, 0.7214 and 0 for Site 1, Site 2 and Site 3 respectively (the Bonferroni-corrected interval for the *p*-value for Site 3 being *p* < 5e− 4).

The maternal MLG of the inoculum was never observed as paternal (*i.e.* detected in spores of harvested fruitbodies;) nor maternal MLGs (*i.e.* detected in gleba of harvested fruitbodies). This respectively rejected hypotheses H1 and H2 (see Fig. 1c).

In conclusion, estimating the relatedness of the spore inoculum to paternal genomes of harvested fruitbodies (hypothesis H3) revealed a not significant relationship at Site 1 and Site 2 but a highly significant at site 3 (r = 0.417, *p*-value < 5e− 4; Table 2). At site 3, truffles produced in traps were fertilized by spores related to those introduced in these traps: In contrast, the spore inoculum never appeared related to the maternal genomes of harvested truffles (hypothesis H4).

## Discussion

**Figure 4:**
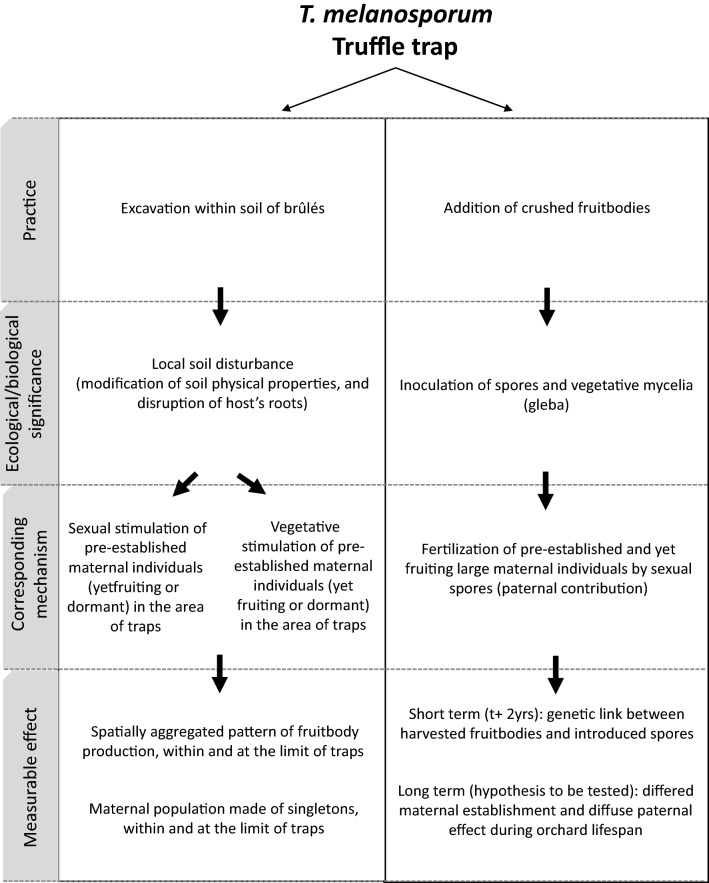
Diagrammatic representation of the mechanisms involved and measurable effects of truffle traps on *T. melanosporum* fruitbody production.

Our results enlighten the biological basis and mechanisms underlying an empirical practice widely used in *T. melanosporum* production. Combining the use of a large dataset assembled by truffle growers with an in situ experiment, we showed that (1) setting truffle traps is an efficient practice to concentrate fruitbody production in small areas in *T. melanosporum* orchards, (2) disturbance effect (traps with no truffle inoculum) alone can concentrate truffle production in traps, probably by stimulating resident mycelia (Fig. 4) and (3) the added inoculum can be directly involved in the mating forming fruitbodies. More precisely, the spores of the added inoculum were observed to act as efficient paternal partners to fertilize resident maternal partners.

### Site depending efficiency of truffle traps

The efficiency of truffle traps was highly variable among sites. In four out of 11 orchards, more than 50% of fruitbodies (in terms of number of fruitbodies) were collected in traps, in approximatively 0.7% of the productive surface of the orchard. Our large-scale survey included a large range of orchard ages (from 3 to 18 years old plantations) and two hosts (*Quercus ilex* and *Q. pubescens*) cultivated in contrasted pedoclimatic conditions. Across this wide range of contexts, our data corroborated two studies which previously evidenced that truffle traps can locally stimulate and concentrate truffle fruitbody production. In a single orchard-case study^6^, reported a significant effect of traps on both the number and the biomass of fruitbodies. Similarly^7^, observed a positive effect of traps on the number of produced fruitbodies at three sites, with a variable effect on produced biomass. The determinants driving the variability of the efficiency of this practice among sites remain unclear, and may include biological (e.g. spore bank richness, established mycelia, host physiology), ecological (e.g. soil physico-chemical conditions, microclimate) and anthropic (practices) parameters.

### Truffle traps provide paternal partners

At Site 3, we demonstrated that some fruitbodies collected in traps were fertilized by individuals related to the spores introduced two years earlier. In contrast, the genetic relationship was not significant in traps positioned at the two other sites. However, this result could be due to lower genetic contrast (and thus, statistical power) of the inoculant with surrounding population since, contrary to Site 3, the added inoculum on these two sites came from local truffle populations (same site for Site 1, and 40 km apart for Site 2). We thus cannot exclude that the contribution of the inoculated spores at these two sites was the same than on Site 3. An alternatively or additional hypothesize is that spores from the soil spore bank may have fertilized truffles collected in traps: such a bank may result from dispersal by the fauna, previous manipulations by growers or even undetected fruitbodies (see below). Indeed, these two sites were characterized by a continuing practice of spore dispersion on soil since host plantation, unlike Site 3 where the soil spore bank was thus certainly less abundant. On the road to truffle domestication, the effect of the dispersion of crushed ascocarps to mastering fruitbody production has been vigorously debated. Our results suggest that the spore bank can be, in some sites, a limiting factor for fruitbody production and that active dispersion by growers has a lasting fertilizing effect in soils at the scale of the truffle orchards (Fig. 4). Our experiment provides the first evidence for the biological basis of this empirical knowledge reported in most grey literature and books devoted to truffle cultivation (*e.g.* for most recent ones^6,7,33,34^ for a recent publications).

It is noteworthy that a majority (68.4%) of collected truffles were not detected by dogs. Missed truffles included all stages (from immature to highly decayed) and all sizes (from 2.7 to 76 g in mass), suggesting that dogs missed some of them at their maturity stage. This result confirms the finding that up to 42% of fruitbodies remain unremoved among multiple managed truffle grounds^35^. These fruitbodies sequestrate undispersed spores which may contribute to explain the extremely high genetic isolation by distance observed in *T. melanosporum* orchards^18,36,37^.

One remaining question concerns the biological determinants of the time required in most orchards to collect fruitbodies in traps (between two to three years; Fig. 2). This may reflect either the time needed for maternal partners to re-establish and be receptive for mating in traps, and/or an incompressible lag phase of sexual spores mobilized as paternal partners to germinate in traps (*e.g.* due to a spore dormancy). Setting up experiments using spores of variable age as source of inoculum (experimentally kept in soil as did^38^ for *Rhizopogon* species) may help to better understand the kinetics of their germination efficiency.

### Truffle traps stimulate the reproduction of established maternal individuals

In truffle traps, maternal genotypes of collected fruitbodies originated from genets which were not genetically related to the introduced crushed fruitbodies (hypotheses H1 & H2; Fig. 1, c.). In other words, the efficiency of truffle traps was not based on the establishment of new maternal genotypes genetically related to the inoculum dispersed in truffle traps, but stemmed from the fertilization of large and perennial maternal individuals pre-established at the immediate proximity of traps.

Remarkably, the analysis of the spatial distribution of these maternal MLGs revealed the presence of several co-occurring genotypes on the same *brûlé*, and even within the same trap (Table S4). At the scale of the *brûlé*, many singletons (*i.e.* maternal MLGs represented by a single ascocarp) co-occurred with large maternal genets extending all over the *brûlé* (Fig. 3c). This co-occurrence of many maternal genotypes of opposite mating types in a majority of productive traps (Figure S2) differ from the patterns previously reported out of the context of truffle traps. In populations previously described, a few large perennial maternal individuals dominate the *brûlé*, producing high numbers of scattered truffles, with spatially close genets carrying identical mating types^18,36^. This result highlights the efficiency of truffle traps to reveal both reproductive and vegetative facets of *T. melanosporum* populations in the field, and suggests that further experiments may take advantage of these tools to finely investigate the unbalanced distribution of maternal and paternal established individuals^18^.

Furthermore, the majority of the ascocarps produced by large maternal individuals were aggregated within traps and/or at the immediate limit of the device (Fig. 3c and Figure S2). This result suggests that a second “truffle trap effect” may be the reproductive stimulation of large maternal individuals pre-established in areas surrounding the traps (*e.g.* Figure 3c and Figure S2). The positive effect of traps on truffle production, and the concentration of mating events at the limit between disturbed and undisturbed soil, (*e.g.* Figure 3c and Figure S2) may be driven by (1) a stimulation of the damaged root system of the host at the limit of traps, with the emergence of secondary roots and mycorrhizal connections and (2) a promoted dynamics of mating between resident maternal and introduced paternal individuals, facilitated by the immediate contact of pre-established mycelia with the added inoculum at the limit of the trap. To support the first point, we note that *T. melanosporum* is a pioneer species^19^, that may efficiently colonize roots after disturbance.

Yet, one striking result of this research is the high number of maternal genotypes which produced only one fruitbody. All but one out of these 30 singletons fruited within truffle traps and or at the immediate limit of the device, and never within the *brûlé* surrounding the traps. Our exhaustive sampling of truffles (systematic opening of traps at the end of the experiment, including the harvest of small truffles not detected by dogs) may have unveiled this structure of *T. melanosporum* population. This result may also suggest that a third “truffle trap effect” may be the sexual activation in the population of vegetative maternal genotypes that were dormant for sexual reproduction, although established in co-occurrence with large ones in the soils of the brûlé (Fig. 4), in a mechanism reminiscent of a “Sleeping beauty effect”^39^. The disturbance may have allowed some dominated individuals to grow up to the level of being able to fruit. This pattern was also observed in truffle traps without inoculum, suggesting that the local soil disturbance in the trap may increase truffle production by stimulating co-occurring dormant maternal genotypes.

## Conclusions

Centuries ago, spore dispersal emerged in spontaneous truffle grounds as a practice to empirically “saw” truffles under established trees (Kieffer^29^). During the twentieth century, based on the generalized use of inoculated plants, planted orchards were propelled as the dominant system of black truffle production. In these highly anthropized ecosystems, ancient practices were reinvented, and more complex designs flourished, including truffle trapping, as attempts to respond to increase truffle production.

In this study, we considered this system as an opportunity to explore the biological bases of a traditional practice developed by truffle growers to intensify *T. melanosporum* production across its natural range. This analysis of truffle growers’ techniques unveiled two elements of the reproduction biology of this highly prized mushroom. First, spore traps may be an adequate response to male shortage in some soils, and further supports an asymmetric system where the stock of ephemeral paternal individuals can be limiting, in contrast with long-lived established maternal partners. As a consequence, in cultivated orchards, spore bank and its renewal by anthropic practices may sustain production in some sites. Second, soil disturbance stimulated the vegetative development of the established population. This finding provides a novel insight into the ecology of the emblematic *T. melanosporum*, by making sense to practices empirically developed during centuries of cultivation.

## Methods

### Dataset 1: Analysis of truffle growers archives

We selected eleven *T. melanosporum* orchards located across the South-West France, from Montpellier (43°44′01.4″N 3°42′13.2″E) to Jonzac (45°27′17.7″N, 0°25′26.9″W; Fig. 2). These sites were selected for (1) the quality of the records of fruitbody production and practices by truffle growers (Table S1), including the detail of inoculations since plantation (amount and frequency of added crushed sporocarps), (2) the use of truffle traps by the owners and the quality of the record from these devices, and (3) the presence of oaks (*Quercus ilex*, *Q. pubescens* and *Q. suber*) as the only hosts tree species. Based on the archives of truffle growers, including a systematic recording of truffle production within and outside traps, we reported at each study site the contribution of truffle traps to the annual fruitbody production of the entire truffle grounds, by using number and/or weight of collected fruitbodies within (P_in_) and outside (P_out_) truffle traps.

### Dataset 2: In situ experiment tracing the inoculation effect

Three orchards located near Angoulème (45°74′35.5″N, − 0°63′78.4″W), Jonzac (45°44′09.8″N, 0°43′96.7″W), and Arles-sur-Tech (42°45′44.9″N, 2°62′89.4″W), hereafter referred to Site 1 to 3 (Fig. 2) were selected for testing both *disturbance effect* and *inoculum effect* on fruitbody production in truffle traps. These sites presented a high fruitbody production and a high P_in_/P_out_ ratio, thus optimum conditions to test mechanisms underlying how truffle traps influence fruitbody production. Host trees were between 5 and 18 years old at the beginning of the experiment (Fig. 2). At each site, we selected three non-adjacent trees (four on Site 3) that displayed a continuous fruitbody production over the three previous years. Under each selected tree, we excavated, at two-thirds of the distance between the tree trunk and the limit of *brûlé* (a vegetation-poor zone that shows the extension mycelia in the soil^40^, eight equidistant truffle traps [20 × 20 cm large × 20 cm deep] as shown in Fig. 3a. Under each tree, two traps were filled with only a mixture of peat and vermiculite (hereafter referred as *non-inoculated controls*) to test for *disturbance effect*. The used mixture was identical to that which is currently applied in commercial orchards. In three other traps, 5 g of crushed material from a single black truffle fruitbody (including its gleba and spores) were added to the previous mixture (hereafter referred as *one mating-type inoculum*). In the three last traps, 5 g of crushed material from two ascocarps with gleba of opposite mating types (hereafter referred as *two mating-type inoculum*) were added to the previous mixture. We added the *two mating-type* condition to accurately test a potential contribution of the gleba (haploid and thus with a single mating type) on future production. As quoted in Introduction, maternal individuals with opposite mating types tend to exclude each other locally (spatial segregation of clusters of individuals of same mating types^26^. Thus, the *two mating-type inoculum* allows us to detect in each trap a maternal contribution by the introduced gleba, despite potential exclusion by pre-installed individuals of the locally dominant mating type in the surrounding. Moreover, it allows us to detect a paternal contribution by the introduced gleba of the mating type opposite to the locally dominant. The eight truffle traps were randomly arranged, so that two repetitions of same modality were always separated by a repetition of another modality (Fig. 3a).

In March 2013, six freshly collected truffles (weighting > 60 g) were molecularly analyzed for the mating type of their gleba as in^18^. On Site 1 and Site 2, the inoculum was made of fruitbodies collected at Site 1. On Site 3, fruitbodies used as inoculum originated from truffle grounds in Sarrion (Spain). In April 2013, truffles traps were installed as explained above (in all, 8 traps × 3 (or 4) trees × 3 sites) and monitored for two years by truffle growers. Harvesting was performed by trained dogs (one different dog per site) checking truffle traps and the surrounding *brûlés* at each visit of the orchard by truffle growers. When dogs detected truffles, a small hole was excavated to collect ascocarps without disturbing the trap further. At the end of January, 2015, all truffle traps were completely excavated, remnant truffles overlooked by dogs were systematically collected (Fig. 3b). Three soil aliquots were collected within all traps and pooled. All truffles and soil aliquots were frozen for subsequent DNA analysis.

### Molecular and genetic analyses

DNA extractions, mating typing and genotyping were done as in^18^. Briefly, DNA was extracted from the gleba and from spores of each fruitbody to get access to the maternal and zygotic DNA, respectively. Simple sequence repeat (SSRs) genotyping was performed using 12 polymorphic markers and the mating-type locus as in^18^. Gleba extracts displaying apparent heterozygous genotypes, likely due to contamination by spore DNA were systematically discarded from further analyses. For each fruitbody, the haploid paternal genotype was then deduced by subtracting the haploid maternal genotype from the zygotic diploid genotype. This data set was used for relatedness estimations. We discarded from all further analysis the marker me11, which displayed more than 39% missing data, as well as all samples with missing data for at any locus.

### Multilocus genotypes comparisons

Based on the 11 remaining SSRs and the mating-type (Table S5 and Figure S2), MLGs were identified on all maternal and paternal haploid genomes using GenClone v.2.0^41^, and the probability that MLGs represented more than once resulted from independent events of sexual reproduction was calculated (*P*_*Sex*_^41,42^). On each site, clonal diversity was measured as R = (G − 1)/(N − 1) according to^43^, where N is the number of fruitbodies and G the number of MLGs. For testing whether the gleba of the inoculated fruitbody contributed, either paternally (H1) or maternally (H2) to the harvested fruitbodies (Fig. 1c), the inoculated maternal MLG was compared to the paternal and maternal MLG of the harvested fruitbodies.

### Relatedness estimation

For testing whether the spores of the inoculum, which carry many distinct haploid MLGs due to meiosis, had paternal or maternal contribution(s) to the harvested fruitbodies (H3; Fig. 1c), we used relatedness estimation.

For testing whether spores of the inoculum had a paternal contribution, an individual relatedness estimate to the spore inoculum was computed for each paternal genome detected in truffle traps. Relatedness *r* here describes the expected frequency E[p_offpat] of each allele in a given genome, E[p_offpat] = p_pop + r * (p_inoc − p_pop), where p_pop is the allele frequency in the local population (here estimated from the glebas of other truffles collected under the focal tree), and p_inoc is the frequency of the allele in the inoculum. Thus, p_offpat takes values 0 or 1, and p_inoc takes values 0, 0.5 or 1, except when two fruitbodies were used as inoculum (*two gleba mating types* traps). Thus r = (p_offpat − p_pop)/(p_inoc − p_pop). An individual relatedness estimate for each genome is then obtained by summing over alleles and loci the observed values of the numerator and denominator in this expression. A population-level estimate is further obtained by summing numerators and denominators over the paternity events in each population.

To test whether such estimates are compatible with the hypothesis that the paternal individuals are not from the inocula, we obtained the distribution of population-level relatedness estimates by simulating samples under this hypothesis: paternal genotypes were randomly simulated according to alleles frequencies in the *local population*. For each population, 10,000 samples were simulated, and *p*-values were estimated as the proportion of simulations with higher population-level relatedness with inocula than the observed one. Confidence intervals for these *p*-values were computed from the binomial distribution for 10,000 draws, and Bonferroni-corrected over the three populations.

For testing whether spores of the inoculum had a maternal contribution (H4, Fig. 1c), we estimated the relatedness of the locally used spore inoculum to each maternal genome detected in truffle traps (deduced from the gleba), and we confronted it to simulated samples as previously but with one modification: if the focal fruitbody was harvested in a trap inoculated with the inoculum A1, all genomes of truffles from traps inoculated with the same inoculum (A1 or A1 + A2 + A3, see Fig. 3c.) were discarded from the estimation of p_pop.

### Assessment of *T. melanosporum* mycelium concentration in truffle traps

On Sites 1, 2 and 3, soil samples were collected in all traps and in the surrounding brûlés at harvesting date (January, 2015). In collected soils, total DNA was extracted and quantified as in^19^. Briefly, after sieving and homogenizing soil collected in each trap and from out of the *brûlés*, aliquots (10 g) were analyzed as follows. After extraction with the kit Power Soil (MoBio Laboratories, Carlsbad, CA, USA), the extra-radical mycelium of *T. melanosporum* was quantified using quantitative Taqman™ PCR (qPCR) with the primers and probe described in^44^. Triplicate real-time PCR were performed on each sample using the same concentration of primer and the same thermocycling program as in^19^. Standards were prepared using fresh immature *T. melanosporum* ascocarp, and a standard curve was generated for each site by plotting serial tenfold dilutions against corresponding initial amount of ascocarp. Absolute quantification of mycelium biomass of *T. melanosporum* was expressed in mg of mycelium per g of soil.

### Statistical analyses

Statistics were done using R version 4.0.4^45^.

*Effect of truffle traps on fruitbody production*—The contribution of truffle traps to the overall production of orchards was assessed by (1) data mining of truffle growers’ archives (Dataset 1) and (2) comparing the density of truffles harvested in traps (expressed in number of truffles per m^2^ per orchard; for each sampled tree, traps correspond to an investigated soil surface of *s* = 8 × 0.2 x 0.2 = 0.32 m^2^) with the density measured within surrounding brûlés (Dataset 1). On Dataset2, at each site, the area occupied by brûlés was evaluated by measuring in the field the surface of soil devoid of vegetation consecutively to spontaneous *T. melanosporum* brûlé.

Fruitbody production under different conditions (i.e. non-inoculated controls *versus* one gleba mating type traps *versus* two gleba mating type traps) were compared using generalized linear mixed models with negative binominal family and log link (R, spam package^46^). The full model included the logarithm of the sampled area as offset to account for variations in this sampled area, interactions of trap-modality effects with site effect. Formal likelihood ratio tests are based on one-step deletions from this full model, applied to subsets of the data relevant for each hypothesis tested. Additional bootstrap tests (1000 iterations) were run to correct any bias in small sample likelihood ratio tests.

*Concentrations of T. melanosporum mycelium in soil*—Similarly as above, the *inoculum effect* on mycelium concentrations was compared using generalized linear mixed models with Gamma log family.

### Plant material

The use of plants in the present study complies with international, national and/or institutional guidelines. All permissions to collect *T. melanosporum* fruitbodies in truffle orchards were obtained. The formal identification of biological material used in the study (*T. melanosporum* fruitbodies) was undertaken by F. Richard and E. Taschen. Voucher specimens of all collected fruitbodies have been deposited in the Centre d’Ecologie Fonctionnelle et Evolutive herbarium in Montpellier (France).

### Ethical approval

All co-authors approve the ethical statement regarding the submitted manuscript.

### Consent to participate

All co-authors consent to participate to the research and agree with the content of the submitted manuscript. All authors reviewed and submitted manuscript.

## Supplementary Information

Supplementary Information 1.

Supplementary Information 2.

## Data Availability

The datasets will be made available on reasonable request upon demand to the corresponding author. The genetic polymorphism datasets generated during the current study are available in the Data INRAE repository, https://doi.org/10.15454/7RCNNI”.
