# Supplementary material for: Efficiency of the traditional practice of traps to stimulate black truffle production, and its ecological mechanisms
Source: Sci Rep. 2022 Sep 28;12:16201. doi: 10.1038/s41598-022-19962-3 (PMC9519532; doi:10.1038/s41598-022-19962-3)
Supplement: Supplementary file 1 — Supplementary Information 1. [file 41598_2022_19962_MOESM1_ESM.pptx]

## Slide 1
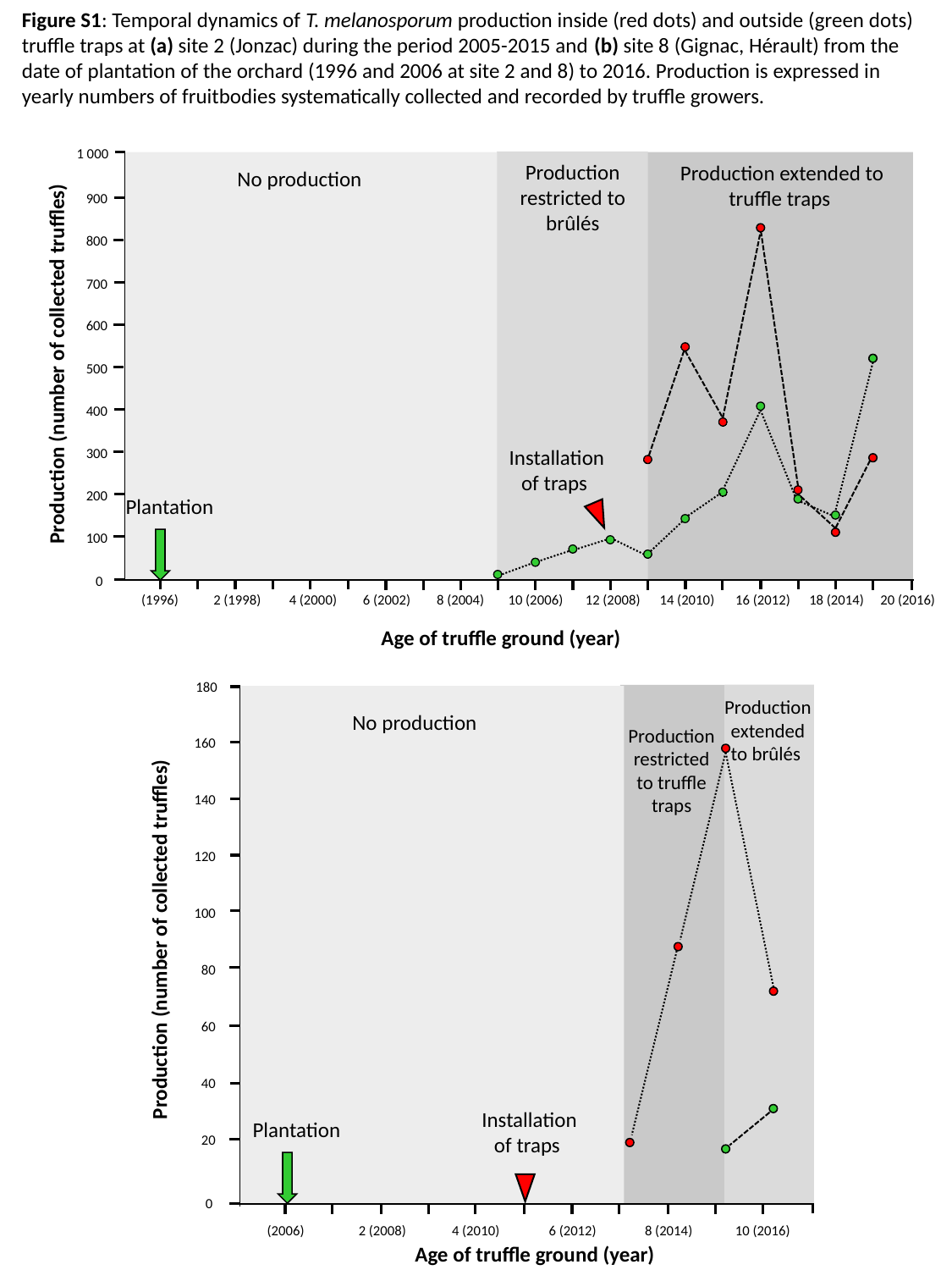

Figure S1: Temporal dynamics of T. melanosporum production inside (red dots) and outside (green dots) truffle traps at (a) site 2 (Jonzac) during the period 2005-2015 and (b) site 8 (Gignac, Hérault) from the date of plantation of the orchard (1996 and 2006 at site 2 and 8) to 2016. Production is expressed in yearly numbers of fruitbodies systematically collected and recorded by truffle growers.
1 000
Production restricted to brûlés
Production extended to truffle traps
No production
900
800
700
600
Production (number of collected truffles)
500
400
Installation
of traps
300
200
Plantation
100
0
(1996)
2 (1998)
4 (2000)
6 (2002)
8 (2004)
10 (2006)
12 (2008)
14 (2010)
16 (2012)
18 (2014)
20 (2016)
Age of truffle ground (year)
180
Production extended to brûlés
No production
Production restricted to truffle traps
160
140
120
100
Production (number of collected truffles)
80
60
40
Installation
of traps
Plantation
20
0
(2006)
2 (2008)
4 (2010)
6 (2012)
8 (2014)
10 (2016)
Age of truffle ground (year)

## Slide 2
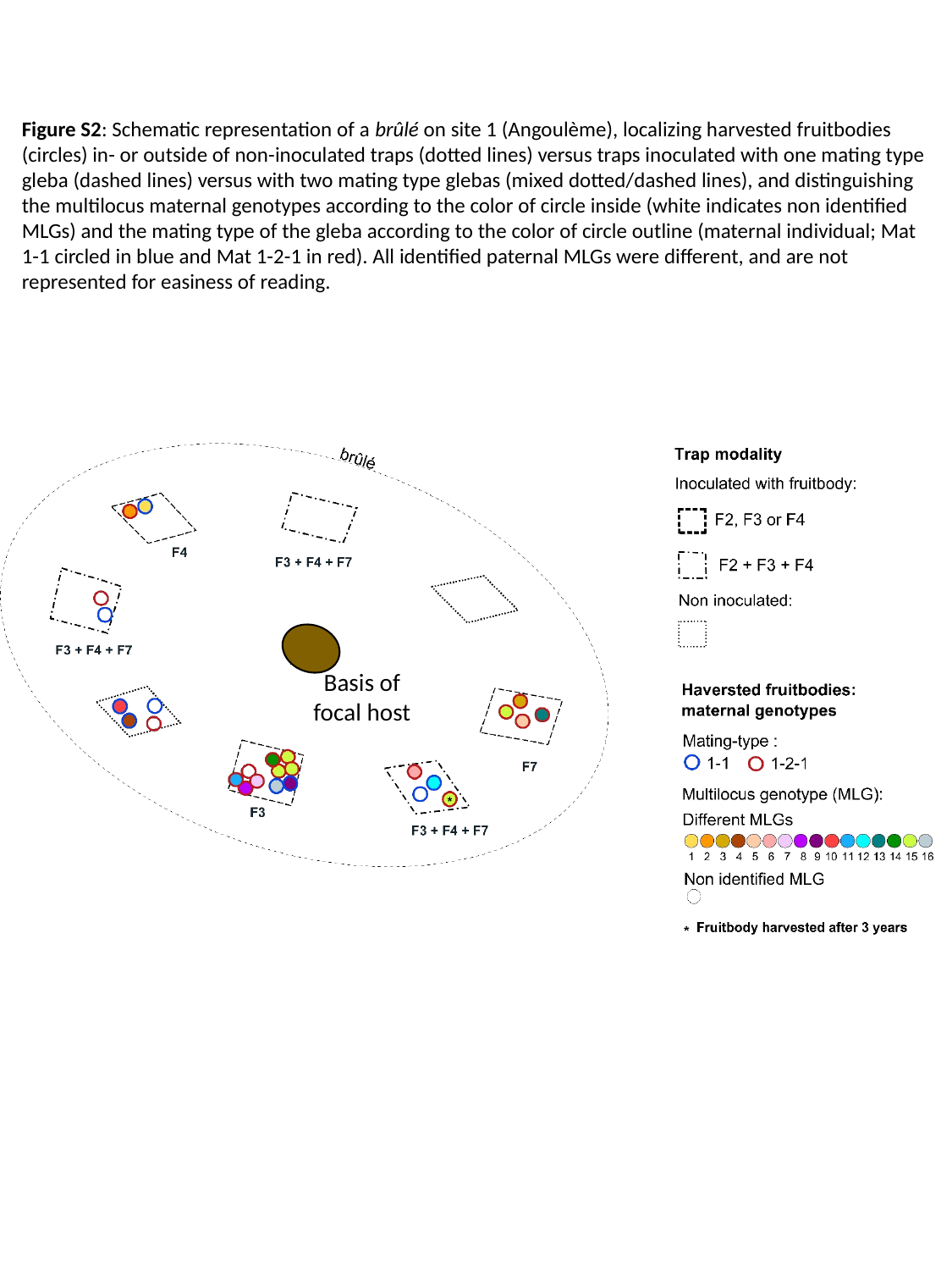

Figure S2: Schematic representation of a brûlé on site 1 (Angoulème), localizing harvested fruitbodies (circles) in- or outside of non-inoculated traps (dotted lines) versus traps inoculated with one mating type gleba (dashed lines) versus with two mating type glebas (mixed dotted/dashed lines), and distinguishing the multilocus maternal genotypes according to the color of circle inside (white indicates non identified MLGs) and the mating type of the gleba according to the color of circle outline (maternal individual; Mat 1-1 circled in blue and Mat 1-2-1 in red). All identified paternal MLGs were different, and are not represented for easiness of reading.
Basis of
focal host

## Slide 3
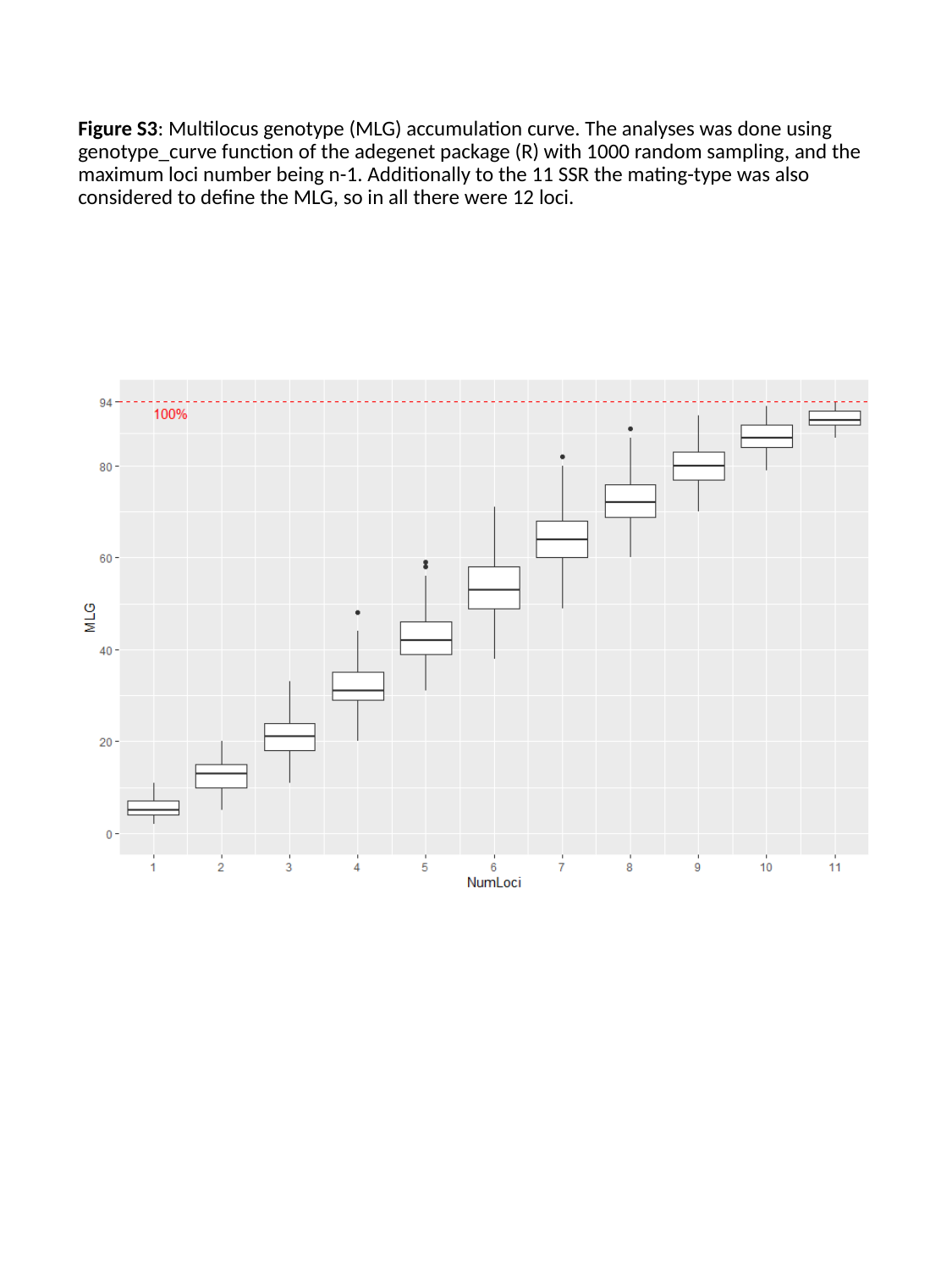

# Figure S3: Multilocus genotype (MLG) accumulation curve. The analyses was done using genotype_curve function of the adegenet package (R) with 1000 random sampling, and the maximum loci number being n-1. Additionally to the 11 SSR the mating-type was also considered to define the MLG, so in all there were 12 loci.
