# Supplementary material for: Efficiency of the traditional practice of traps to stimulate black truffle production, and its ecological mechanisms
Source: Sci Rep. 2022 Sep 28;12:16201. doi: 10.1038/s41598-022-19962-3 (PMC9519532; doi:10.1038/s41598-022-19962-3)
Supplement: Supplementary file 2 — Supplementary Information 2. [file 41598_2022_19962_MOESM2_ESM.docx]

**Supplementary Material**

**Table S1**: Characteristics of T. *melanosporum* orchards, design of truffle traps positioned by truffle growers in the field since plantation, and detail of fruitbody production in and outside the traps at 11 studied sites. The data were systematically collected by truffle growers.


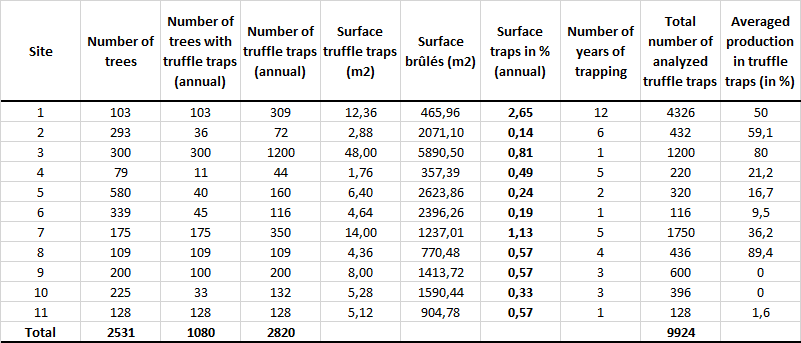


**Table S2**: Fruitbody harvest on each experimental tree at three experimental sites, with the detail of collected fruitbodies in and outside the traps, corresponding surfaces (m^2^) and the related density of production.

|  | **Number of fruitbodies** | | | | | |  | **Surface (m^2^)** | |  | **Density of fruitbodies (/m^2^)** | | | |
| --- | --- | --- | --- | --- | --- | --- | --- | --- | --- | --- | --- | --- | --- | --- |
| **Site/Tree species** | **All traps** | **Non inoculated control traps** | **1-gleba mating type traps** | **2-gleba mating type traps** | **All inoculated traps** | **The rest of the *brûlé*** |  | **Traps (m²)** | **brûlés** |  | **Non inoculated control traps** | **1-gleba mating type** | **2-gleba mating type** | **All inoculated traps** |
| **1 (Angoulême)** |  |  |  |  |  |  |  |  |  |  |  |  |  |  |
| *Q. ilex* | 25 | 4 | 16 | 5 | 21 | 0 |  | 0,32 | 4,52 |  | 50,00 | 133,33 | 41,67 | 87,50 |
| *Q. ilex* | 4 | 2 | 0 | 2 | 2 | 1 |  | 0,32 | 4,52 |  | 25,00 | 0,00 | 16,67 | 8,33 |
| *Q. ilex* | 4 | 0 | 2 | 2 | 4 | 6 |  | 0,32 | 4,52 |  | 0,00 | 16,67 | 16,67 | 16,67 |
| **Total** | **33** | **6** | **18** | **9** | **27** | **7** |  | 0,96 | 13,57 |  | **25,00** | **50,00** | **25,00** | **37,50** |
| **2 (Jonzac)** |  |  |  |  |  |  |  |  |  |  |  |  |  |  |
| *Q. pubescens* | 4 | 0 | 0 | 4 | 4 | 1 |  | 0,32 | 7,07 |  | 0,00 | 0,00 | 33,33 | 16,67 |
| *Q. pubescens* | 28 | 6 | 12 | 10 | 22 | 3 |  | 0,32 | 7,07 |  | 75,00 | 100,00 | 83,33 | 91,67 |
| *Q. pubescens* | 1 | 0 | 0 | 1 | 1 | 0 |  | 0,32 | 7,07 |  | 0,00 | 0,00 | 8,33 | 4,17 |
| **Total** | **33** | **6** | **12** | **15** | **27** | **4** |  | 0,96 | 21,21 |  | **25,00** | **33,33** | **41,67** | **37,50** |
| **3 (Arles sur Tech)** |  |  |  |  |  |  |  |  |  |  |  |  |  |  |
| *Q. pubescens* | 1 | 1 | 1 | 0 | 1 | 3 |  | 0,32 | 7,07 |  | 12,50 | 8,33 | 0,00 | 4,17 |
| *Q. ilex* | 3 | 0 | 1 | 2 | 3 | 3 |  | 0,32 | 7,07 |  | 0,00 | 8,33 | 16,67 | 12,50 |
| *Q. ilex* | 0 | 0 | 0 | 0 | 0 | 2 |  | 0,32 | 7,07 |  | 0,00 | 0,00 | 0,00 | 0,00 |
| *Q. ilex* | 24 | 2 | 11 | 11 | 22 | 5 |  | 0,32 | 7,07 |  | 25,00 | 91,67 | 91,67 | 91,67 |
| **Total** | **28** | **3** | **13** | **13** | **26** | **13** |  | 1,28 | 28,27 |  | **9,38** | **27,08** | **27,08** | **27,08** |

**Table S3**: Summary of the t-values from the glmm testing for differences in the number of fruitbodies on the *brûlé* *versus* in the different trap modalities. Differences in surfaces were included into the model. The model was written as following, using the spam package: fitme(truffles ~offset(log(area))+trap*site, family=negbin(), data=harvest) ; with “truffle”= number of fruitbodies, “area”= surface, “trap”= trap modality (with the control non-inoculated trap as intercept), “site”= experimental site.

**Table S4**: Maternal genetic diversity on each experimental tree at three experimental sites, with the numbers of successfully genotyped fruitbodies on the total amount of sampled fruitbodies within parentheses.

**Table S5**: Detailed presentation of the 12 SSR polymorphic markers used in the study. *Na: Number of observed alleles and **He: Expected heterozygosity, calculated 1091 samples including ascocarps, ECM and others samples. SSR name with « me »: Riccioni et al., 2008. (https://doi.org/10.1111/j.1469-8137.2008.02560.x). SSR name with « Tm”: Murat et al., 2011. (https://doi.org/10.1016/j.fgb.2010.10.007).
